# Supplementary material for: Impact of Lung Compliance on Neurological Outcome in Patients with Acute Respiratory Distress Syndrome Following Out-of-Hospital Cardiac Arrest
Source: J Clin Med. 2020 Feb 14;9(2):527. doi: 10.3390/jcm9020527 (PMC7073731; doi:10.3390/jcm9020527)
Supplement: Supplementary file 1 [file jcm-09-00527-s001.pdf]

Impact of lung compliance on neurological outcome in patients with acute respiratory distress syndrome following out-of-hospital cardiac arrest

Table S1. Comparisons of mechanical ventilator parameters in the ARDS patients. We divided parameters into two groups by neurologic outcomes. Because trends of each variable changed variously during three days, we categorized maximum, median, and minimum for three days. Most of tidal volumes and minute ventilations showed no differences between two groups. Meanwhile, compliances after ROSC and day-1 had higher in good outcome group than that of poor group.

Characteristics	Total (n = 119)	Poor outcome (n = 96)	Good outcome (n = 23)	p-value
FiO_2 (%)				
After ROSC	100.0 (80.0 – 100.0)	100.0 (90.0 – 100.0)	100.0 (60.0 – 100.0)	0.028
D1 maximum	70.0 (50.0 – 100.0)	70.0 (50.0 – 100.0)	100.0 (43.0 – 100.0)	<0.001
D1 minimum	80.0 (60.0 – 100.0)	60.0 (50.0 – 70.0)	40.0 (40.0 – 75.0)	<0.001
D1 median	65.0 (50.0 – 100.0)	60.0 (50.0 – 80.0)	60.0 (40.0 – 60.0)	0.059
D2 maximum	60.0 (50.0 – 90.0)	50.0 (45.0 – 70.0)	35.0 (35.0 – 50.0)	0.001
D2 minimum	50.0 (40.0 – 75.0)	55.0 (50.0 – 70.0)	40.0 (35.0 – 50.0)	0.003
D2 median	50.0 (40.0 – 80.0)	50.0 (40.0 – 60.0)	35.0 (32.5 – 45.0)	0.003
D3 maximum	45.0 (40.0 – 60.0)	50.0 (40.0 – 60.0)	35.0 (35.0 – 55.0)	0.012
D3 minimum	50.0 (40.0 – 65.0)	50.0 (40.0 – 65.0)	40.0 (35.0 – 50.0)	0.003
D3 median	50.0 (40.0 – 65.0)	40.0 (35.0 – 65.0)	40.0 (35.0 – 50.0)	0.003
D4	50.0 (40.0 – 62.5)	50.0 (45.0 – 70.0)	50.0 (35.0 – 50.0)	0.016
D5	50.0 (40.0 – 60.0)	50.0 (40.0 – 55.0)	50.0 (40.0 – 55.0)	0.795
D6	45.0 (40.0 – 50.0)	50.0 (40.0 – 50.0)	50.0 (37.5 – 55.0)	0.968
PaO_2 (mmHg)				
After ROSC	86.0 (68.4 – 115.8)	86.0 (70.4 – 115.8)	77.1 (62.5 – 137.4)	0.287
D1 maximum	113.0 (88.0 – 143.0)	109.0 (94.0 – 131.9)	139.5 (107.1 – 156.8)	0.374
D1 minimum	69.0 (57.5 – 83.9)	64.0 (58.0 – 77.0)	73.5 (63.2 – 82.5)	0.230
D1 median	88.3 (72.0 – 110.5)	83.3 (69.0 – 95.0)	102.3 (94.9 – 114.2)	0.448
D2 maximum	102.9 (86.0 – 133.3)	106.0 (90.8 – 143.0)	109.7 (90.8 – 124.4)	0.418
D2 minimum	69.0 (60.0 – 78.8)	72.5 (64.0 – 79.6)	80.8 (64.8 – 93.4)	0.097
D2 median	81.0 (71.4 – 91.3)	80.0 (72.0 – 88.0)	90.8 (82.1 – 101.5)	0.216
D3 maximum	97.1 (84.3 – 108.0)	101.0 (81.0 – 110.0)	97.1 (83.0 – 114.0)	0.807
D3 minimum	72.0 (60.0 – 78.0)	70.0 (58.0 – 79.7)	76.1 (61.8 – 81.5)	0.120
D3 median	82.8 (69.0 – 90.8)	79.4 (65.0 – 91.2)	85.9 (71.8 – 94.6)	0.085
D4	87.6 (74.0 – 92.3)	87.0 (74.0 – 95.0)	95.5 (87.4 – 102.2)	0.417
D5	90.4 (70.0 – 101.0)	88.5 (70.0 – 101.0)	95.5 (88.9 – 102.6)	0.263
D6	81.0 (70.8 – 96.0)	81.0 (69.0 – 95.8)	83.9 (75.0 – 137.4)	0.660
$PaCO_2$ (mmHg)				

After ROSC	54.0 (40.0 – 73.0)	57.0 (43.5 – 76.0)	46.0 (32.6 – 60.5)	0.005
D1 maximum	40.5 (34.4 – 49.1)	43.4 (39.0 – 52.0)	38.3 (32.6 – 44.3)	0.027
D1 minimum	44.0 (37.0 – 52.0)	44.1 (39.0 – 53.3)	46.5 (41.9 – 55.1)	0.950
D1 median	40.9 (35.7 – 47.4)	42.0 (38.0 – 46.0)	43.7 (33.8 – 47.6)	0.362
D2 maximum	37.8 (33.0 – 42.0)	41.0 (33.0 – 44.8)	40.5 (34.8 – 44.6)	0.085
D2 minimum	41.0 (35.5 – 47.5)	38.2 (35.7 – 46.0)	41.0 (35.7 – 46.9)	0.216
D2 median	40.8 (36.0 – 46.8)	43.0 (36.0 – 47.0)	50.0 (42.4 – 53.4)	0.304
D3 maximum	39.0 (35.0 – 42.9)	38.0 (35.0 – 41.0)	39.5 (33.6 – 43.7)	0.510
D3 minimum	41.5 (38.4 – 46.0)	43.0 (39.0 – 46.0)	41.8 (38.9 – 47.7)	0.932
D3 median	41.7 (38.0 – 44.5)	41.8 (38.0 – 45.2)	41.4 (41.2 – 44.5)	0.137
D4	40.6 (36.8 – 45.9)	41.0 (36.9 – 48.0)	43.3 (37.4 – 47.7)	0.925
D5	40.0 (35.1 – 45.9)	40.0 (32.8 – 49.0)	37.0 (34.5 – 43.4)	0.336
D6	39.1 (33.4 – 44.0)	40.0 (34.0 – 48.0)	33.6 (31.5 – 43.1)	0.298
PEEP (cm H_2O)				
After ROSC	5.0 (4.0 – 8.0)	5.0 (4.0 – 8.0)	5.0 (4.0 – 7.0)	0.638
D1 maximum	6.0 (4.0 – 8.0)	8.0 (5.0 – 10.0)	5.5 (4.8 – 8.5)	0.174
D1 minimum	6.0 (5.0 – 10.0)	9.0 (6.0 – 12.0)	6.5 (4.0 – 11.0)	0.387
D1 median	8.0 (5.0 – 10.0)	8.0 (5.0 – 12.0)	5.0 (4.0 – 10.5)	0.030
D2 maximum	8.0 (5.0 – 10.0)	6.0 (5.0 – 10.0)	5.0 (4.8 – 8.5)	0.421
D2 minimum	8.0 (5.0 – 10.0)	8.0 (5.0 – 10.0)	5.0 (4.8 – 8.5)	0.326
D2 median	7.0 (5.0 – 10.0)	6.0 (5.0 – 10.0)	5.0 (4.0 – 10.0)	0.398
D3 maximum	6.0 (5.0 – 8.0)	6.0 (5.0 – 10.0)	5.0 (4.0 – 6.5)	0.183
D3 minimum	6.0 (5.0 – 8.0)	6.0 (5.0 – 10.0)	5.0 (4.0 – 6.0)	0.074
D3 median	6.0 (5.0 – 8.0)	6.0 (5.0 – 10.0)	5.0 (4.0 – 6.3)	0.172
D4	6.0 (4.0 – 8.0)	6.0 (5.0 – 10.0)	5.0 (4.0 – 8.5)	0.704
D5	6.0 (5.0 – 8.0)	6.0 (5.0 – 8.0)	5.0 (4.8 – 6.0)	0.357
D6	5.0 (4.0 – 6.8)	5.0 (5.0 – 8.0)	5.0 (3.8 – 6.0)	0.160
Inspiratory pressure (cm H_2O)				
After ROSC	15.0 (12.0 – 18.0)	16.0 (14.0 – 18.0)	12.0 (12.0 – 13.5)	<0.001
D1 maximum	14.0 (12.0 – 18.0)	16.0 (12.0 – 20.0)	12.0 (11.5 – 14.0)	<0.001
D1 minimum	15.0 (12.0 – 18.0)	15.0 (14.0 – 20.0)	13.0 (11.5 – 16.5)	<0.001
D1 median	14.0 (12.0 – 18.0)	14.0 (14.0 – 20.0)	12.0 (11.5 – 16.0)	0.001
D2 maximum	14.0 (12.0 – 17.5)	14.0 (12.0 – 20.0)	15.5 (11.5 – 16.3)	0.431
D2 minimum	14.0 (14.0 – 16.0)	14.0 (12.0 – 16.0)	15.5 (13.0 – 16.3)	0.724
D2 median	14.0 (12.0 – 18.0)	14.0 (12.0 – 16.0)	15.5 (11.5 – 18.5)	0.280
D3 maximum	14.0 (12.0 – 16.0)	14.0 (12.0 – 16.0)	15.0 (14.0 – 16.5)	0.916
D3 minimum	14.0 (12.0 – 16.0)	14.0(12.0 – 16.0)	14.5 (13.8 – 18.0)	0.879
D3 median	140. (12.0 – 16.0)	12.0 (12.0 – 16.0)	14.5 (13.5 – 17.0)	0.620
D4	14.0 (12.0 – 16.0)	14.0 (12.0 – 16.0)	14.0 (13.5 – 16.5)	0.588
D5	14.0 (12.0 – 16.0)	20.0 (18.0 – 24.0)	19.0 (16.8 – 20.5)	0.489
D6	14.0 (12.0 – 16.0)	19.0 (18.0 – 23.0)	18.5 (14.8 – 20.3)	0.570
P_{plat} (cm H_2O)				

After ROSC	21.0 (17.3 – 25.8)	21.0 (18.0 – 22.0)	17.0 (16.0 – 20.0)	<0.001
D1 maximum	22.0 (18.0 – 26.0)	23.0 (19.0 – 28.0)	18.0 (16.3 – 22.5)	<0.001
D1 minimum	23.0 (18.5 – 28.0)	24.0 (21.0 – 29.0)	19.0 (16.3 – 28.5)	0.002
D1 median	22.0 (18.0 – 27.0)	24.0 (21.0 – 28.0)	17.0 (15.5 – 26.5)	<0.001
D2 maximum	23.0 (18.5 – 27.5)	23.0 (18.0 – 26.0)	20.5 (16.3 – 25.3)	0.122
D2 minimum	22.0 (20.0 – 26.0)	22.0 (20.0 – 28.0)	20.5 (17.8 – 25.3)	0.201
D2 median	22.0 (19.0 – 26.0)	20.0 (19.0 – 25.0)	21.0 (16.3 – 27.0)	0.214
D3 maximum	21.0 (18.0 – 24.0)	21.0 (17.0 – 26.0)	21.0 (19.0 – 22.0)	0.192
D3 minimum	21.0 (18.0 – 24.0)	22.0 (18.0 – 26.0)	20.0 (18.5 – 22.5)	0.083
D3 median	21.0 (18.0 – 26.0)	20.0(17.0 – 28.0)	20.0 (18.8 – 21.8)	0.481
D4	20.5 (18.0 – 24.3)	21.0 (18.0 – 26.0)	19.5 (18.3 – 20.5)	0.170
D5	14.0 (12.0 – 16.0)	20.0 (18.0 – 24.0)	19.0 (16.8 – 20.5)	0.143
D6	19.0 (17.3 – 21.0)	19.0 (18.0 – 23.0)	18.5 (14.8 – 20.3)	0.269
Respiratory rate				
After ROSC	20.0 (18.0 – 22.0)	20.0 (18.0 – 22.0)	18.0 (15.0 – 20.0)	0.013
D1 maximum	20.0 (16.0 – 24.0)	20.0 (16.0 -24.0)	16.0 (13.5 – 19.0)	<0.001
D1 minimum	20.0 (18.0 – 24.0)	20.0 (17.0 – 23.0)	22.0 (20.0 – 22.0)	0.002
D1 median	20.0 (16.0 – 24.0)	22.0 (18.0 – 24.0)	17.0 (13.5 – 24.0)	0.004
D2 maximum	20.0 (16.0 – 22.0)	18.0 (16.0 – 22.0)	18.0 (14.0 – 21.0)	0.051
D2 minimum	22.0 (20.0 – 24.0)	20.0 (18.0 – 20.0)	18.0 (15.0 – 22.0)	0.287
D2 median	20.0 (15.5 – 24.0)	20.0 (16.0 – 22.0)	16.0 (13.5 – 24.0)	0.476
D3 maximum	18.0 (16.0 – 20.0)	20.0 (16.0 – 20.0)	16.0 (14.0 – 19.0)	0.283
D3 minimum	18.0 (14.0 – 22.0)	18.0 (14.0 – 24.0)	19.0 (14.0 – 20.5)	0.328
D3 median	18.0 (14.0 – 22.0)	19.0 (14.0 – 20.0)	17.0 (14.0 – 20.5)	0.894
D4	18.0 (14.0 – 20.0)	18.0 (14.0 – 20.0)	14.0 (14.0 – 17.5)	0.475
D5	18.0 (14.0 – 22.0)	18.0 (14.0 – 22.0)	16.0 (14.0 – 22.0)	0.522
D6	16.0 (14.0 – 18.0)	18.0 (15.0 – 20.0)	16.0 (13.5 – 16.0)	0.143
Tidal volume (ml)				
After ROSC	427.0 (367.0 – 497.0)	420.0 (360.0 – 489.5)	458.0 (395.5 – 545.0)	0.053
D1 maximum	434.0 (363.0 – 498.0)	416.0 (362.0 – 493.0)	453.0 (419.0 – 456.5)	0.150
D1 minimum	429.0 (355.0 – 486.0)	427.0 (368.0 – 450.0)	474.0 (410.0 – 488.5)	0.591
D1 median	422.0 (368.5 – 468.0)	417.0 (386.0 – 475.0)	430.0 (426.5 – 470.0)	0.387
D2 maximum	424.5 (373.5 – 485.5)	417.0 (386.0 – 495.0)	422.0 (421.5 – 436.0)	0.543
D2 minimum	440.5 (371.8 – 476.0)	449.0 (393.0 – 476.0)	444.0 (443.5 – 445.0)	0.837
D2 median	424.5 (348.8 – 478.5)	411.0 (367.0 – 462.0)	449.0 (439.5 – 514.5)	0.036
D3 maximum	432.0 (384.0 – 478.0)	400.0 (380.0 – 452.0)	474.0 (447.0 – 609.5)	0.438
D3 minimum	420.0 (684.0 – 466.0)	430.0 (389.0 – 467.0)	420.0 (415.5 – 438.5)	0.613
D3 median	419.5 (362.8 – 461.5)	417.0 (384.0 – 449.0)	411.0 (405.5 – 452.5)	0.544
D4	429.0 (386.0 – 471.0)	412.0 (363.0 – 457.0)	425.0 (422.5 – 504.0)	0.813
D5	419.0 (370.5 – 475.8)	406.0 (380.0 – 476.0)	440.0 (418.0 – 476.0)	0.428
D6	437.0 (370.0 – 466.0)	432.0 (370.0 – 466.0)	442.0 (415.0 – 496.0)	0.851
Minute volume (L/min)				

After ROSC	8.2 (7.6 – 10.4)	8.8 (7.7 – 10.3)	8.8 (6.5 – 10.7)	0.609
D1 maximum	8.63 (7.37 – 10.9)	8.7 (6.6 – 9.8)	7.4 (6.8 – 7.9)	0.271
D1 minimum	9.0 (7.0 – 10.5)	9.4 (7.6 – 11.0)	7.7 (6.3 – 9.0)	0.105
D1 median	8.7 (7.2 – 10.1)	8.0 (6.7 – 10.0)	6.7 (6.5 – 9.5)	0.002
D2 maximum	8.3 (6.7 – 9.7)	8.5 (6.6 – 9.5)	8.4 (7.8 – 8.4)	0.216
D2 minimum	8.5 (6.9 – 9.7)	8.6 (7.5 – 9.2)	8.4 (7.7 – 8.7)	0.387
D2 median	8.2 (6.5 – 9.7)	8.3 (6.4 – 9.6)	8.0 (7.1 – 11.0)	0.987
D3 maximum	7.8 (6.3 – 10.2)	8.7 (6.3 – 11.4)	9.7 (7.7 – 10.2)	0.553
D3 minimum	8.4 (6.6 – 10.1)	8.6 (7.2 – 10.7)	9.2 (8.0 – 10.5)	0.733
D3 median	7.4 (5.7 – 9.7)	7.7 (5.5 – 10.1)	9.1 (7.9 – 9.1)	0.852
D4	7.9 (5.9 – 9.6)	7.1 (4.9 – 9.1)	8.2 (7.3 – 8.9)	0.599
D5	7.9 (6.8 – 9.2)	7.2 (5.6 – 8.5)	7.3 (6.4 – 8.4)	0.818
D6	8.0 (6.2 – 10.0)	8.1 (6.1 – 10.7)	7.0 (6.6 – 7.3)	0.648
Compliance (ml/cm H_2O)				
After ROSC	29.7 (21.8 – 36.9)	27.5 (21.6 – 34.0)	38.6 (33.1 – 46.2)	< 0.001
D1 maximum	30.9 (23.7 – 35.9)	30.0 (26.1 – 32.1)	32.9 (32.5 – 35.3)	<0.001
D1 minimum	27.4 (21.9 – 33.8)	27.4 (21.6 – 33.9)	28.8 (27.6 – 32.4)	0.009
D1 median	29.8 (21.6 – 35.3)	29.0 (23.7 – 33.6)	35.3 (33.6 – 35.5)	0.002
D2 maximum	30.6 (23.3 – 35.1)	31.9 (27.5 – 37.1)	30.0 (27.4 – 32.5)	0.282
D2 minimum	29.8 (22.7 – 34.5)	32.8 (28.1 – 36.1)	29.6 (27.9 – 30.6)	0.357
D2 median	29.3 (23.0 – 34.3)	31.1 (28.6 – 33.4)	29.0 (28.8 – 33.2)	0.036
D3 maximum	29.9 (25.4 – 34.9)	32.3 (27.1 – 34.9)	33.9 (31.9 – 40.2)	0.399
D3 minimum	29.4 (23.8 – 33.2)	30.7 (28.9 – 34.7)	27.4 (26.4 – 28.7)	0.720
D3 median	28.9 (23.6 – 34.5)	32.0 (26.2 – 35.2)	27.4 (26.1 – 28.0)	0.846
D4	30.3 (24.9 – 34.3)	29.9 (26.3 – 33.8)	30.4 (30.2 – 31.4)	0.820
D5	28.2 (23.2 – 33.8)	31.7 (25.1 – 35.9)	31.4 (29.9 – 34.0)	0.263
D6	30.8 (24.9 – 37.8)	30.4 (26.8 – 33.2)	36.8 (32.3 – 38.1)	0.487

Data are presented as median with interquartile ranges.

Abbreviations: ARDS = acute respiratory distress syndrome; ROSC = return of spontaneous circulation; D = day.

TABLE S2. Baseline characteristics of the whole study population

Characteristics	Total (n = 246)	No ARDS (n = 127)	ARDS (n = 119)	*P*-value
Age	62.0 (48.8 – 74.0)	56.0 (43.0 – 71.0)	66.0 (55.0 – 78.0)	< 0.001
Male	159 (64.6)	80 (63.0)	79 (66.4)	0.596
Past history				
Acute coronary syndrome	51 (20.7)	25 (19.7)	26 (21.8)	0.694
Arrhythmia	24 (9.8)	11 (8.7)	13 (10.9)	0.668
Congestive heart failure	15 (6.1)	6 (4.7)	9 (7.6)	0.429
Stroke	14 (5.7)	5 (3.9)	9 (7.6)	0.275
Hypertension	88 (35.8)	31 (24.4)	57 (47.9)	< 0.001
Diabetes mellitus	63 (25.6)	18 (14.2)	45 (37.8)	< 0.001
Chronic pulmonary disease	19 (7.7)	7 (5.5)	12 (10.1)	0.233
Chronic kidney disease	32 (13.0)	9 (7.1)	23 (19.3)	0.005
Malignancy	27 (11.0)	15 (11.8)	12 (10.1)	0.689
Characteristics of Cardiac arrest				
Witnessed arrest	184 (74.8)	98 (77.2)	86 (72.3)	0.383
Bystander CPR	169 (68.7)	94 (74.0)	75 (63.0)	0.074
Shockable rhythm	71 (36.4)	49 (49.0)	22 (23.2)	< 0.001
Cardiac cause	117 (47.6)	62 (48.8)	55 (46.2)	0.703
Emergent PCI	28 (11.3)	19 (15.0)	9 (7.6)	< 0.049
Total CPR time (min)	24.0 (11.8 – 37.0)	21.0 (10. – 31.0)	29.0 (14.0 – 41.0)	0.005
SOFA at admission	11.0 (8.0 – 13.0)	8.0 (7.0 – 11.8)	12.0 (11.0 – 15.0)	< 0.001

Data are presented as n (%) or median with interquartile ranges. P<.05 are presented in bold. Abbreviations: ARDS = acute respiratory distress syndrome; PCI = percutaneous coronary intervention; CPR = cardiopulmonary resuscitation; TTM = target temperature management; sBP = systolic blood pressure; dBP = diastolic blood pressure; SOFA = Sequential Organ Failure Assessment.

Table S3-1. Baseline characteristics of the ARDS patients. We included the patients with severe LV dysfunction (LV EF < 30%) proven by echocardiography for evaluating the impact of cardiogenic pulmonary edema on neurologic outcomes (N = 9). Table S2-1, S2-2, S2-3 showed similar trends with main findings in the manuscript.

Characteristics	Total (n = 128)	Poor outcome (n = 104)	Good outcome (n = 24)	p-value
Age	67.0 (56.0 – 77.8)	67.0 (56.0 – 77.0)	66.0 (57.0 – 79.8)	0.876
Male	84 (65.6)	66 (63.5)	18 (75.0)	0.346
Past history				
Previous CA	1 (0.8)	0 (0.0)	1 (4.2)	0.187
Previous MI	7 (5.5)	4 (3.8)	3 (12.5)	0.121
Previous ACS	21 (16.4)	16 (15.4)	5 (20.8)	0.544
Previous PCI	18 (14.1)	13 (12.5)	5 (20.8)	0.329
Stroke	10 (7.8)	8 (7.7)	2 (8.3)	1.000
HTN	62 (48.4)	53 (51.0)	9 (37.5)	0.264
DM	47 (36.7)	40 (38.5)	7 (29.2)	0.485
COPD	13 (10.2)	13 (12.5)	0 (0.0)	0.126
CKD	26 (20.3)	22 (21.2)	4 (16.7)	0.782
LC	3 (2.3)	3 (2.9)	0 (0.0)	0.623
Malignancy	14 (10.9)	12 (11.5)	2 (8.3)	0.740
Characteristics of Cardiac arrest				
In-hospital arrest	11 (8.6)	5 (4.8)	6 (25.0)	0.006
Witnessed arrest	89 (69.5)	71 (68.3)	18 (75.0)	0.627
Bystander CPR	84 (65.6)	69 (66.3)	15 (62.5)	0.812
Shockable rhythm	22 (21.6)	13 (14.8)	9 (64.3)	<0.001
Cardiac cause	58 (45.3)	42 (40.4)	16 (66.7)	0.024
Emergent PCI	9 (7.0)	6 (5.8)	3 (12.5)	0.050
Total CPR time (min)	29.0 (14.3 – 41.0)	31.0 (22.0 – 42.0)	12.0 (4.8 – 21.0)	<0.001
SOFA at admission	12.0 (11.0 – 15.0)	12.0 (11.0 – 15.0)	11.5 (10.5 – 15.0)	0.642
MV duration (day)	7.0 (3.3 – 9.0)	7.0 (2.0 – 9.0)	7.0 (5.0 – 12.3)	0.101

Data are presented as median with interquartile ranges.

Abbreviations: ARDS = acute respiratory distress syndrome; CA = cardiac arrest; MI = myocardial infarction; ACS = acute coronary syndrome; PCI = percutaneous coronary intervention; CHF = chronic heart failure; HTN = hypertension; DM = diabetes mellitus; CPOD = chronic pulmonary obstructive disease; CKD = chronic kidney disease; LC = liver cirrhosis; CPR = cardiopulmonary resuscitation; MV = mechanical ventilator.

Table S3-2. Comparisons of ABGA and mechanical ventilator parameters on admission in the ARDS patients

Parameters	Total (n = 128)	Poor outcome (n = 104)	Good outcome (n = 24)	p-value
pH	7.22 (7.14 – 7.30)	7.22 (7.11 – 7.30)	7.29 (7.22 – 7.38)	0.006
PaO_2 (mmHg)	87.15 (72.0 – 109.8)	84.2 (72.0 – 108.0)	96.5 (77.8 – 105.2)	0.614
$PaCO_2$ (mmHg)	40.9 (35.8 – 48.1)	42.0 (36.0 – 49.0)	39.4 (33.5 – 45.2)	0.361
FiO_2 (%)	60.0 (50.0 – 100.0)	70.0 (60.0 – 100.0)	40.0 (40.0 – 60.0)	< 0.001
P/F ratio	138.4 (98.9 – 198.9)	129.1 (98.6 – 180.0)	206.0 (137.8 – 255.8)	0.003
PEEP (cm H2O)	8.0 (5.0 – 10.0)	8.0 (5.0 – 10.0)	5.0 (4.0 – 9.0)	0.048
Inspiratory pressure (cm H_2O)	14.0 (12.0 – 18.0)	15.0 (12.0 – 20.0)	12.0 (12.0 – 14.0)	< 0.001
Tidal volume (ml)	422.5 (369.3 – 469.3)	427.0 (370.0 – 470.0)	442.0 (401.0 – 496.0)	0.250
Compliance (ml /cm H_2O)	29.8 (21.6 – 35.3)	29.0 (20.8 – 34.6)	35.0 (29.7 – 45.7)	0.002
Minute ventilation (L/min)	8.7 (7.2 – 10.2)	8.9 (7.5 – 10.3)	7.0 (6.3 – 8.6)	0.007

Abbreviations: ABG = arterial blood gas; ROSC = return of spontaneous circulation; ARDS = acute respiratory distress syndrome; PIP = peak inspiratory pressure; PEEP = positive end expiratory pressure; P/F = partial pressure of arterial oxygen / fraction of inspired oxygen.

Table S3-3. Multivariate logistic regression of parameters associated with favorable neurologic outcome

Variables	Univariate analysis			Multivariate analysis		
	OR	95% CI	*p*	Adjusted OR	95% CI	*p*
CPR duration	0.941	0.909 – 0.974	0.001	0.947	0.900 – 0.997	0.039
Age	0.994	0.964 – 1.025	0.702			
Sex	1.727	0.631 – 4.726	0.287			
TTM	0.786	0.265 – 2.333	0.664	1.834	0.105 – 32.105	0.678
Shockable rhythm	1.176	0.959 – 1.441	0.119			
PCI	1.016	0.403 – 2.564	0.973			
Bystander CPR	1.164	0.360 – 3.77	0.799			
Lung compliance						
Maximum	1.045	0.1028 – 1.062	<0.001	1.052	1.019 – 1.086	0.002
Median	1.047	1.025 – 1.069	<0.001	1.056	1.024 – 1.089	0.005
Minimum	1.064	1.035 – 1.094	<0.001	1.079	1.034 – 1.127	<0.001

Abbreviations: OR = odds ratio; CI = confidence interval; CPR = cardiopulmonary resuscitation; TTM = target temperature management; PCI = percutaneous coronary intervention.
